# Effect of platelet-rich plasma on angiogenic and regenerative properties in patients with critical limb ischemia

**DOI:** 10.1016/j.reth.2025.01.008

**Published:** 2025-02-06

**Authors:** Hamid Tanzadehpanah, Sima Nobari, Ava Jalalian Hoseini, Farzaneh Ghotbani, Mohsen Mehrabzadeh, Jamal Jalili shahri, Amirreza Alipour, Mohsen Sheykhhasan, Hamed Manoochehri, Susan Darroudi, Hanie Mahaki

**Affiliations:** a Antimicrobial Resistance Research Center, Basic Science Research Institute, Mashhad University of Medical Sciences, Mashhad, Iran; b Metabolic Syndrome Research Center, Mashhad University of Medical Sciences, Mashhad, Iran; c Deputy of Health, Iran University of Medical Science, Tehran, Iran; d Research Committee, Mashhad University of Medical Sciences, Mashhad, Iran; e Vascular and Endovascular Surgery Research Center, Mashhad University of Medical Sciences, Mashhad, Iran; f Cellular and Molecular Research Center, Qom University of Medical Sciences, Qom, Iran; g The Persian Gulf Marine Biotechnology Research Center, The Persian Gulf Biomedical Sciences Research Institute, Bushehr University of Medical Sciences, Bushehr, Iran

**Keywords:** Platelet-rich plasma, Angiogenic factor, Growth factor, Critical limb ischemia, Peripheral arterial disease

## Abstract

Platelet-rich plasma (PRP) is a promising regenerative therapy due to its simplicity, clinical application, safety, and ability to promote angiogenesis. It utilizes various angiogenic growth factors in platelets, including platelet-derived growth factor (PDGF), transforming growth factor-β (TGF-β), insulin-like growth factor-1 (IGF-1), vascular endothelial growth factor (VEGF), and epidermal growth factor (EGF), which are integral to the tissue repair.

Critical limb ischemia (CLI) is a major symptom of peripheral arterial disease (PAD), and PRP therapy aims to improve blood circulation to the distal limb through the development of blood vessels. This review focuses on the extensive research on the molecular mechanisms of PRPs in treating CLI. A comprehensive search was conducted on Web of Science, PubMed, Google Scholar, and Scopus to find studies published during PRP therapy in critical limb ischemia up to June 2024. Current studies reveal that PRP composition varies by case, affecting preparation methods, storage duration, storage methods, and interaction with other materials. PRP-derived growth factors have shown promising results in treating CLI, but well-controlled human research is scarce despite positive animal studies.

## 1 Introduction

Peripheral arterial disease (PAD) is a chronic vascular disease, and CLI is defined as the end stage of it. It contributes to cardiovascular morbidity and mortality, affecting approximately 230 million individuals globally [1,2]. Critical limb ischemia (CLI) is a medical disorder characterized by chronic ischemic at-rest pain, ulcers, or gangrene in lower limbs due to objectively verified arterial occlusive disease [2,3]. The prevalence of CLI in the US is estimated to reach 2 million annually. CLI Patients are more prone to multifocal disease and have variable immunological responses. They are at a higher risk of experiencing subsequent myocardial infarction, stroke, and vascular mortality [4].

CLI is a significant medical concern due to its detrimental impact on quality of life and weak prognosis for limb salvage and survivability [5]. CLI patients in classes IV according to Fontaine classification and classes 6 according to Rutherford have a serious problem with long-term hospitalization, high levels of morbidity, high costs, and a low quality of life [6]. Wound healing processes require self-repair mechanisms to restore the unity of mucosal and skin barriers. The hypoxic/ischemic environment of CLI leads to highly dysregulated processes, resulting in prolonged ulceration tissue [7].

CLI patients are identified until the disease progresses, highlighting the need for biomarkers for stratification and personalized medical treatments [8]. Over the past two decades, regenerative medicine treatments have been proposed to address the unmet clinical needs of CLI patients. Gene/protein therapy using growth factors (GFs) and stem cell-based therapy were initially introduced to promote reparative angiogenesis in affected muscles [9]. Small interfering RNA (siRNA) is a potent method for suppressing gene expression in mammalian cells [[10], [11], [12]]. The study found that silencing SHP-1 siRNA reduced TNF's effectiveness in preventing KDR/flk-1 tyrosine phosphorylation by VEGF, in cases of hindlimb ischemia [13]. Zheng and et al. have developed a novel treatment for kidney ischemia/reperfusion injury by silencing the C5a receptor gene with C5aR siRNA [14]. Song et al. have demonstrated that siRNA can effectively silence C3, preventing complement activation and protecting against renal ischemia-reperfusion [15].

Platelet-rich plasma (PRPs) contain biomarkers and cytokines such as platelet-derived growth factor (PDGF), basic fibroblast growth factor (bFGF), vascular endothelial growth factor (VEGF), insulin-like growth factor-1 (IGF-1), transforming growth factor-β (TGF-β), and others that stimulate cell production of collagen and extracellular matrix, promoting angiogenesis, mitogenesis, and macrophage activation [16].

**Fig. 1:**
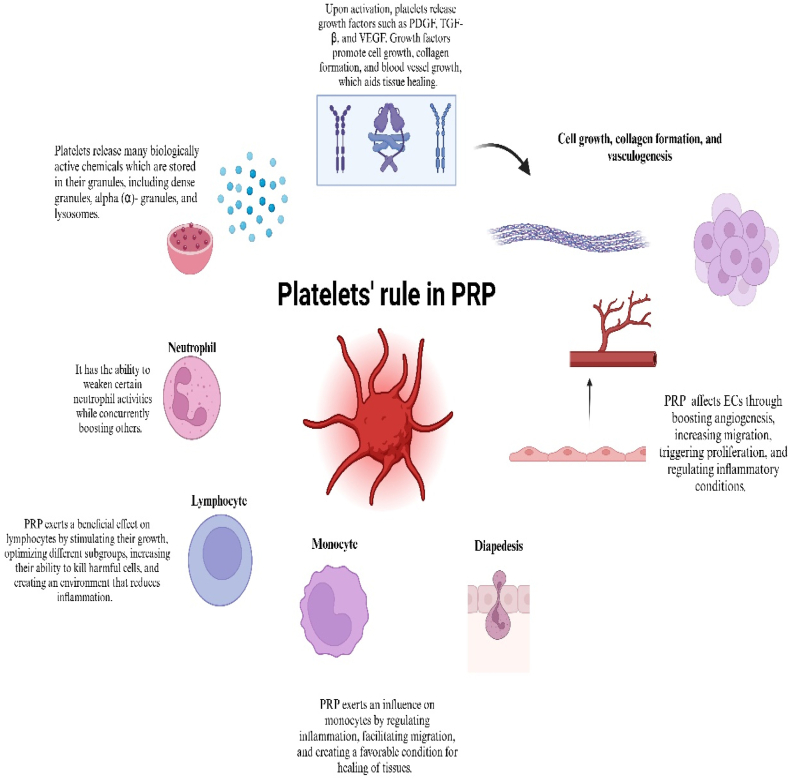
**Therapeutic potential of platelets cells.** The mechanism of action of platelets, which are rich in growth factors and interact with other immune cells by secreting different cytokines, is what gives PRP its therapeutic effects. It promotes angiogenesis and collagen deposition, increases epithelial and endothelial cell regeneration, and speeds up the healing process. Platelets were used to treat ulcers and wounds much more quickly.

Furthermore, PRP can release antibacterial agents, mitigate local inflammation, and avert wound infection. It has gained popularity among clinics due to its ease of utilization, favorable biological safety, noninvasive characteristics, high growth factor concentration and low immunogenicity [17,18]. We have presented a summary of the latest trials that evaluated the feasibility of PRP therapy in CLI based on the properties mentioned. We are exploring this issue for the first time (Fig. 1).

## 2 Selection and search strategy of the literature

This narrative review search was performed using Web of Science, PubMed, Google Scholar, and Scopus. We searched for studies published during PRP therapy in CLI (up to June 2024). The keywords such as (“Angiogenic factors”, OR “interested angiogenic factor name”), AND (“Platelet-rich plasma”, OR “PRP”), AND “Critical limb ischemia”, OR “CLI”) with mesh term, were used. All studies ranging from original articles (in vitro and in vivo studies), clinical trial studies, and review studies were used in this review, provided that they were published in English and had undergone peer review.

## 3 Classification of PRP in clinical applications

**Fig. 2:**
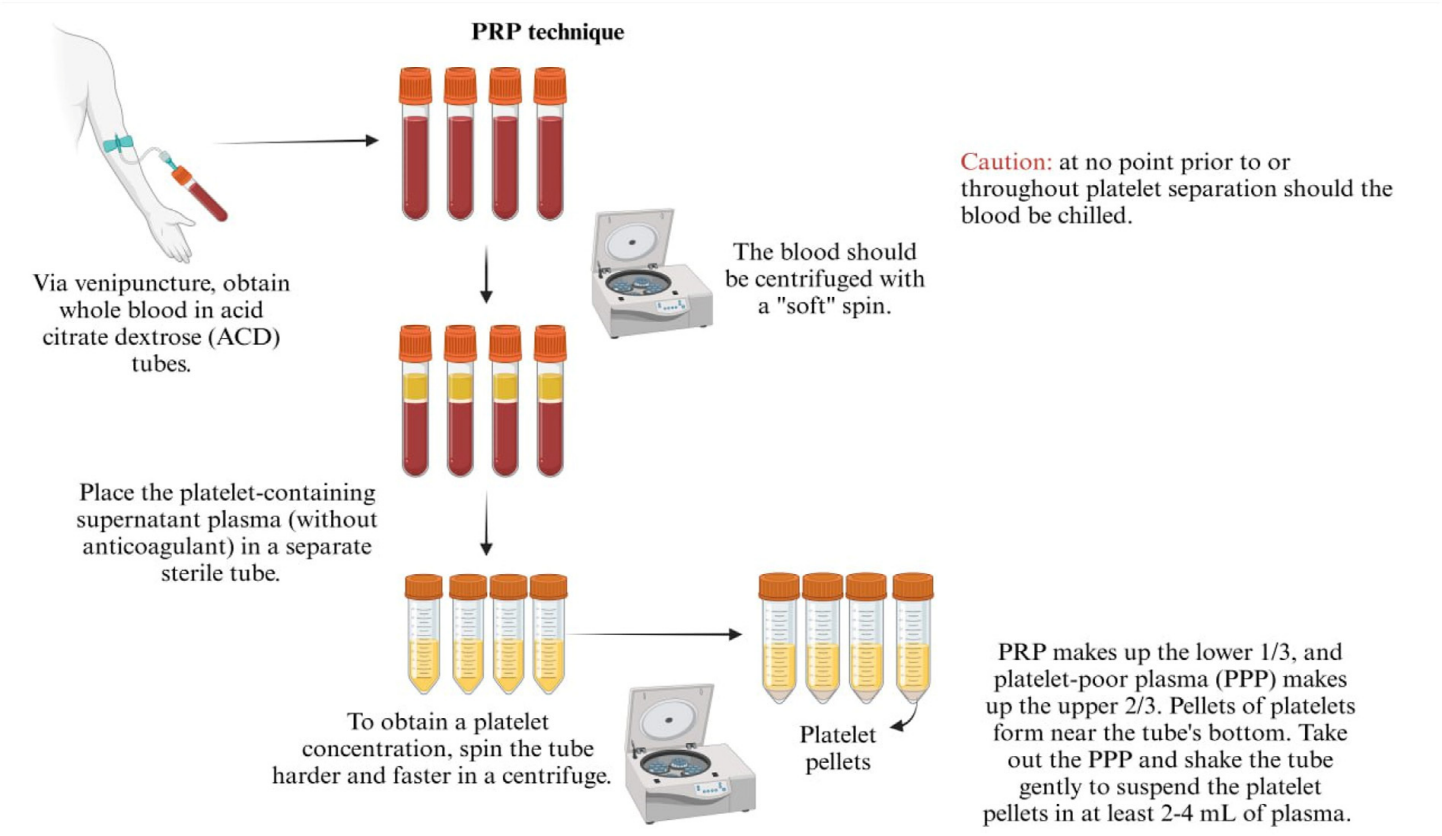
**Procedures for the preparation of platelet-rich plasma.** The platelet-rich plasma is prepared through differential centrifugation, which involves adjusting the acceleration force to sediment specific cellular constituents based on their specific gravity. Initially, the red blood cells (RBC) are separated through centrifugation, followed by a second centrifugation to concentrate platelets, which are suspended in the smallest final plasma volume. The first spin is conducted at 200G for 12 min, and the second spin is conducted at 1600G for 8 min without any manipulation. Lastly, a 20G long needle is inserted to aspirate the PPP with the assistance of an 18G needle (used as a guide).

Plasma protein from autologous peripheral blood platelets is autologous PRP. The variety of PRP-production methods has produced many products. These products exhibit significant variations in several key aspects [19]. Firstly, the volume of whole blood harvested during PRP preparation varies by technique. Secondly, some methods involve the inclusion or exclusion of white blood cells (WBCs) in the final PRP product. Thirdly, some techniques can externally activate platelets, whereas others do not [20]. Overall, these differences highlight the diverse nature of PRP products resulting from the utilization of distinct techniques (Fig. 2).

Dohan Ehrenfest et al. classified PRP types using the data found in scientific literature. The researchers divided PRP into four groups based on leukocyte and fibrin presence [21]. Their research categorized the PRPs described in the current literature into four groups: pure platelet-rich plasma (P-PRP), leukocyte- and platelet-rich plasma (L-PRP); pure platelet-rich fibrin (P-PRF); and leukocyte- and platelet-rich fibrin (L-PRF) [22,23].

### 3.1 Pure platelet-rich plasma (P-PRP)

P-PRP is distinguished by its high concentration of platelets with minimal leukocyte presence, achieved through specialized centrifugation. This formulation minimizes inflammation while providing a concentrated supply of growth factors, including PDGF, VEGF, and TGF-β, which are critical for tissue repair, angiogenesis, and cell proliferation. P-PRP is most effective in conditions where inflammation management is crucial, such as chronic wounds in CRL and degenerative conditions like osteoarthritis. Supporting these results, some studies found that P-PRP is beneficial in scenarios where reducing inflammation is advantageous, such as in chronic wounds. Studies show P-PRP’s concentrated growth factors promote cell proliferation and angiogenesis, key for tissue repair, making it a valuable choice for patients needing controlled inflammation and quick healing [24,25].

### 3.2 Pure platelet-rich fibrin (P-PRF)

P-PRF forms a fibrin matrix that naturally sustains growth factor release over an extended period. This dense structure serves as a scaffold for cell migration and tissue repair, releasing essential growth factors like PDGF, VEGF, and TGF-β in a controlled manner. P-PRF is particularly suited for applications requiring prolonged growth factor delivery, such as oral and bone regeneration, where the fibrin matrix supports gradual tissue repair [26].

### 3.3 Leukocyte-and platelet-rich plasma (L-PRP)

L-PRP combines both platelets and leukocytes, supporting immune modulation and tissue repair. L-PRP’s leukocytes release additional cytokines and growth factors (e.g., IL-6, IL-8, and IGF), promoting inflammation and enhancing healing at sites susceptible to infection. This makes L-PRP a suitable option for wound types that benefit from a robust inflammatory response, such as musculoskeletal injuries, where both immune activation and tissue regeneration are advantageous [27].

### 3.4 Leukocyte-and platelet-rich fibrin (L-PRF)

L-PRF features a dense fibrin network and a high leukocyte content, extending growth factor release while contributing immune support through cytokine release. L-PRF is advantageous in wound healing applications requiring both immune regulation and sustained regeneration, as in periodontal healing or orthopedic procedures, where both infection control and gradual tissue repair are essential. The presence of leukocytes in L-PRF promotes a balanced inflammatory response, which is beneficial for controlling infection and supporting regeneration [27,28].

Selecting the optimal platelet-rich product for wound healing in critical limb ischemia involves a balance between the wound’s inflammatory state, the need for cellular regeneration, and the desired immune response. Each P-PRP, P-PRF, L-PRP, and L-PRF offers distinct therapeutic benefits based on its cellular composition, growth factor release profile, and method of application [29].

In summary, P-PRP and P-PRF are most suitable for reducing inflammation and delivering growth factors over time, ideal for chronic or low-inflammation wound settings such as CRL. Conversely, L-PRP and L-PRF are more effective in environments needing enhanced immune support and infection control, where both leukocytes and a sustained fibrin matrix contribute to optimized tissue repair. This differentiation allows for tailored therapeutic approaches that maximize wound healing potential across a range of clinical applications [30].

## 4 Mechanisms of PRP in the repair of CLI

CLI is caused by insufficient arterial blood flow to the limbs. This condition often reduces tissue blood flow during rest, lowering quality of life. The most common treatment of CLI involves arterial revascularization to enhance blood flow.

[31,32].

### 4.1 PRP in promoting growth factors release for repair of CLI

Activated platelets secrete growth factors that make PRP effective. Growth factors, which regulate chemotaxis, mitogenesis, and differentiation, are mostly stored in thrombocyte alpha-granules [33]. Active platelets release growth factors that affect adjacent mesenchymal and epithelial cells. These growth factors stimulate these cells to migrate, divide, and produce collagen and matrix [34].

PRP contains several types of growth factors that play a significant role in the healing process. These include PDGF, TGF-β, VEGF, EGF, IGF, and FGF (Table 1) [35]. Given the widespread application of the PRP method across various clinical domains, numerous in vitro and in vivo studies have been conducted to evaluate its effectiveness. Furthermore, a series of in vitro and in vivo studies investigating the PRP treatment in patients with CLI were identified (Table 2). These studies investigated the effectiveness of PRP in treating ischemic conditions. The findings suggest that in most cases, PRP promotes the proliferation of vascular cells, improving oxygen delivery and blood flow to ischemic areas. However, some studies reported no significant differences between PRP and conventional treatment methods for ischemia.

**Table 1:** PRP-based growth factors in wound healing.

Growth Factors	Cell Sources	Tissue	Species	Function	References
EGF	Platelets, monocytes, macrophages	Blood, skin Blood Musculoskeletal	Human HUVECs Human	Enhances the production of cytokines by mesenchymal and epithelial cells, while also promoting the proliferation and specialization of epithelial cells.	(86–91)
PDGF	Platelets, macrophages, smooth muscle cells, endothelial cells	Blood, skin, bone Skin Connective tissue/epithelium, muscle, blood	Human Mice Mice, Pigs	Leads to an upregulation in collagen expression, cellular proliferation, fibroblast chemotaxis, proliferative activities, and macrophage activation.	(87, 88, 91–98)
FGF	Platelets, mesenchymal cells, Chondrocytes, osteoblasts, Macrophages	Blood, skin Blood, skin Muscle, blood	Human Mice Mice and Pigs	Facilitates the development, proliferation, and differentiation of chondrocytes and osteoblasts, enhances the growth of mesenchymal cells, and regulates cell survival and migrations.	(91, 95, 98–100)
TGF	Macrophages, Keratinocytes, T Lymphocytes	Muscle Musculoskeletal, bone Muscle, skin, blood	Rats Human Human	Enhances angiogenesis and immune cell migration, regulates collagen type 1 production and collagenase secretion, promotes endothelial cell migration and neovascularization, and suppresses macrophage and lymphocyte proliferation.	(86–88, 90, 91, 93, 97, 101–103)
HGF	Platelets, mesenchymal cells	Skin, blood Skin	Mice Human	Controls the growth and movement of epithelial and endothelial cells, cell movement, and invasion of the extracellular matrix, and consequently enhances the repair of the epithelium and the formation of new blood vessels.	(95, 100, 104)
IGF-1	Platelets, fibroblasts, plasma, Osteoblasts, endothelial cells, Bone matrix	Blood, bone, skin Tendons, skin Connective tissue/epithelium, skin	Human Rabbits Pig	Stimulate fibroblasts, promoting cellular development, facilitate bone production by stimulating the proliferation and differentiation of osteoblasts.	(86, 87, 91, 94, 105–107)
VEGF	Platelets, endothelial cells macrophages, Keratinocytes	Blood skin Skin, blood Blood, muscle	BALB/c nude Mice Rats Human, Mice, Pigs	Induces angiogenesis, attracts immune cells, promotes the movement and division of endothelial cells, and increases the permeability of blood vessels.	(87, 88, 91, 93, 95, 98, 108, 109)
TNF	Macrophages, T-lymphocytes, Mast cells	Skin, blood Skin, Blood	Human Rats Humans, Dog, Horse	Controls migration of monocytes, the growth of fibroblasts, stimulation of macrophages, and the formation of new blood vessels.	(86, 93, 110–113)

Abbreviations: Epidermal growth factor (EGF), Fibroblast growth factor (FGF), Hepatocyte growth factor (HGF), Insulin-like growth factor-1 (IGF-1), Platelet-derived growth factor (PDGF), Transforming growth factor-β (TGF-B), Tumor necrosis factor (TNF), Vascular endothelial growth factor (VEGF).

**Table 2:** Studies of platelet-rich plasma (PRP) treatment in patients with critical limb ischemia.

Author	Study type	Species/Cell culture	Intervention	Evaluation technique	Etiology	Factor	Mechanism	Result
Shyamal Chandra Bir MBBS, 2009 (114)	In vivo, In vitro	Mice	PRP	Laser Doppler, anti-vWF, anti-SMA antibody	The right femoral artery	CD34^+^, SDF-1α, PDGF-BB, VEGF, bFGF, IGF-1	PRP stimulates endothelial cell proliferation.	PRP's continuous release enhanced blood flow in the ischemic region
Liangliang du, 2022 (115)	A Parallel RCT	Human	Combination of autologous platelet-rich gel, conventional treatment, and traditional Chinese medicine	Laboratory variables: platelets, hemoglobin (Hb), albumin, and HbA1c	DFU	platelets, Hb, albumin, HbA1c	PRP promotes endothelial cell proliferation.	The rate of regeneration was significantly increased and the laboratory variable didn’t show valuable variation
William C. Krupski, 1991 (116)	A randomized, prospective, double-blind, placebo-controlled study	Human	Topical PDWHF	ABI, transcutaneous oxygen tension	Refractory lower extremity wounds	–	PDWHF promotes angiogenesis, increasing the production of fibroblast cells	No notable disparity in the pace of healing, size of the healing region, or ABI between the two groups
Marwa Ahmed, 2017 (117)	Prospective comparative study	Human	Autologous platelet gel, thrombin, and calcium chloride with an antiseptic ointment dressing	The study compares the rate of healing with the rate of wound infection	Clean chronic DFU	HbA1C Hb Platelets, Leucocytes	Thrombin degranulates platelets	The use of autologous platelet gel is more successful than local antiseptic dressing
Ignacio Gallo, 2013 (118)	In vivo	Ewes	Platelet gel vs. normal saline	Hematoxylin-eosin staining: Angiogenesis assessment Measurement of factor VIII	Myocardial infarction	PRGF, factor VIII	Stimulate cell division and the formation of new blood vessels in sheep hearts that previously suffered a cardiac attack	PRP treatment increased neovascularization and factor VIII in PRGF-treated myocardia
Barbara Hargrave, 2012 (119)	In vivo, In vitro	Female white rabbits/H9c2 cells	NsPEF-activated PRP vs. normal saline	Nanosecond pulse electric, infarct staining, and its dimension measurement, H9c2 cell culture, flow cytometry	Myocardial infarction	ROS	PRP aids in protecting the heart by decreasing the production of ROS and regulating mitochondria in the region affected by ischemia and reperfusion.	PRP treatment reduced ROS production and mitochondrial depolarization in H9c2 cells and reduce infarcts effects
Soh Nishimoto, 2013 (120)	In-vivo	Male white rabbits	Bm-PRP and pb-PRP	Macroscopic observation of the wounds, dyed cell tracing via DiI-labeled cells	Persistent ischemic limb lesion	–	Enhances delivery of bone marrow cells to the ischemic heart and neovascularization and cardiac muscle preservation	The bm-PRP resulted in quicker wound healing in an ischemic limb, but pb-PRP did not demonstrate any significant improvement
Volodymyr Goshchynsky, 2020 (121)	In-vivo	Human	Transluminal revascularization and PRP vs. traditional post-amputation wound care	Siemens angiography and 3D CT scan	Fourth-grade critical lower limb ischemia	ІL - 1β, ІL- 4, TNF- α, IF-γ	Collateral growth stimulation	PRP treatment enhances blood flow to the distal regions of the extremities
Maki Morita, 2023 (122)	In vivo, In vitro	Male Lewis rats, male Balb/c-nu/nu mice	PRP injection and ADSC transplantation	RT-PCR and Western Blot, eNOS production assessment, laser Doppler blood flow analysis, and tube formation assay	Intractable hypoxic conditioned ulcers	VEGF-A, bFGF, TGFβ1, HGF, NO	ADSCs promote angiogenesis via the secretion of angiogenic factors	PRP stimulated the proliferation of ADSCs obtained from rats and decreased NO production
Yaqiong Zhu, 2022 (123)	In vivo, In vitro	Sprague–Dawley rats	Nerve microtissue graft and PRP	Modified Tarlov score for neurological function analysis, CEUS examination of vein grafts, and RT-PCR	Repairing tibial nerve defects	NGF-β, VEGF, BDNF, and GDNF	PRP via plentiful supply of stem cells and a diverse range of natural growth factors help to make up for the lack of stem cells in the nerve bridges	The Micro-T + PRP group and autograft groups exhibited the best nerve repair

Abbreviation: platelet-poor plasma (PPP); solution form of PRP (PRP-sol); sustained release form of PRP (PRP-sr); Von Willebrand factor (vWF); anti-smooth muscle actin (SMA) antibody; Insulin-like Growth Factor 1 (IGF-1); Diabetic foot ulcer (DFU); platelet-derived wound healing factors (PDWHF); ankle-brachial index (ABI); platelet-rich plasma (PRP); nanosecond pulsed electric fields (nsPEF); Reactive Oxygen Species (ROS); bone marrow aspirate (bm-PRP); peripheral blood (PB-PRP); Vascular endothelial growth factor (VEGF); fibroblast growth factor (FGF); transforming growth factor-beta (TGF-β); granulocyte-macrophage colony-stimulating factor (GM-CSF); adipose-derived stem cells (ADSCs); Micro-T (Microtissues); Schwann cells (SCs); High-frequency ultrasound and contrast-enhanced ultrasonography (CEUS); Real-time reverse transcription polymerase chain reaction (RT-PCR).

#### 4.1.1 Platelet-derived growth factor (PDGF)

During blood clotting, platelets release PDGF from their alpha granules, which stimulates cell processes including revascularization and collagen production to heal wounds [36,37]. Platelets secrete PDGF, which attracts monocytes to heal wounds. These monocytes differentiate into macrophages and release PDGF, which recruits more immune cells to the injury site [38,39].

Moreover, PDGF stimulates the synthesis of key constituents of the extracellular matrix, such as fibronectin and hyaluronic acid. PDGF also triggers the movement and proliferation of vascular smooth muscle cells, fibroblasts, and monocytes towards the damaged area in the context of ischemic inflammation [40]. By drawing monocytes to vascular injury, PDGF helps to curtail pro-inflammatory actions and promotes inflammation resolution through the autocrine feedback inhibition of platelet aggregation. These results indicate that PDGF has a crucial function in controlling the recruitment of immune cells and the occurrence of inflammation throughout the process of tissue repair and regeneration [[41], [42], [43]].

#### 4.1.2 Transforming growth factors (TGFs)

PRP contains several types of TGFs, including TGF-β1 and β2. These elements are regarded as crucial growth and differentiation factors that have a key role in the regeneration of connective tissue [44]. TGFβ1 and β2 are known to stimulate the proliferation and differentiation of cells involved in connective tissue regeneration, such as fibroblasts and osteoblasts. Moreover, they are also involved in extracellular matrix production, collagen synthesis, and promoting angiogenesis in the affected area [[45], [46], [47]]. Research indicates that TGF-β impacts endothelial cells differently in vitro and in vivo. In vitro, TGFβ inhibits endothelial cells, whereas in vivo, it stimulates them. The angiogenic activity of TGFβ is thought to be mediated by interactions with other cellular components, such as macrophages, rather than directly affecting endothelial cells [48].

#### 4.1.3 Vascular endothelial growth factor (VEGF)

VEGF, as a powerful protein, stimulates angiogenesis by the formation of blood vessels. Lab experiments show that VEGF greatly increases endothelial cell growth and proliferation, which are crucial to blood vessels. It does this by binding to specific transmembrane receptors on endothelial cells, including FLT-1, FLK-1, and neuropilin-1 [49]. Fluids and chemicals can pass blood vessel walls because VEGF improves vascular permeability. However, prolonged blood vessel permeability can cause tissue swelling, such as edema in ischemia [50].

**Fig. 3:**
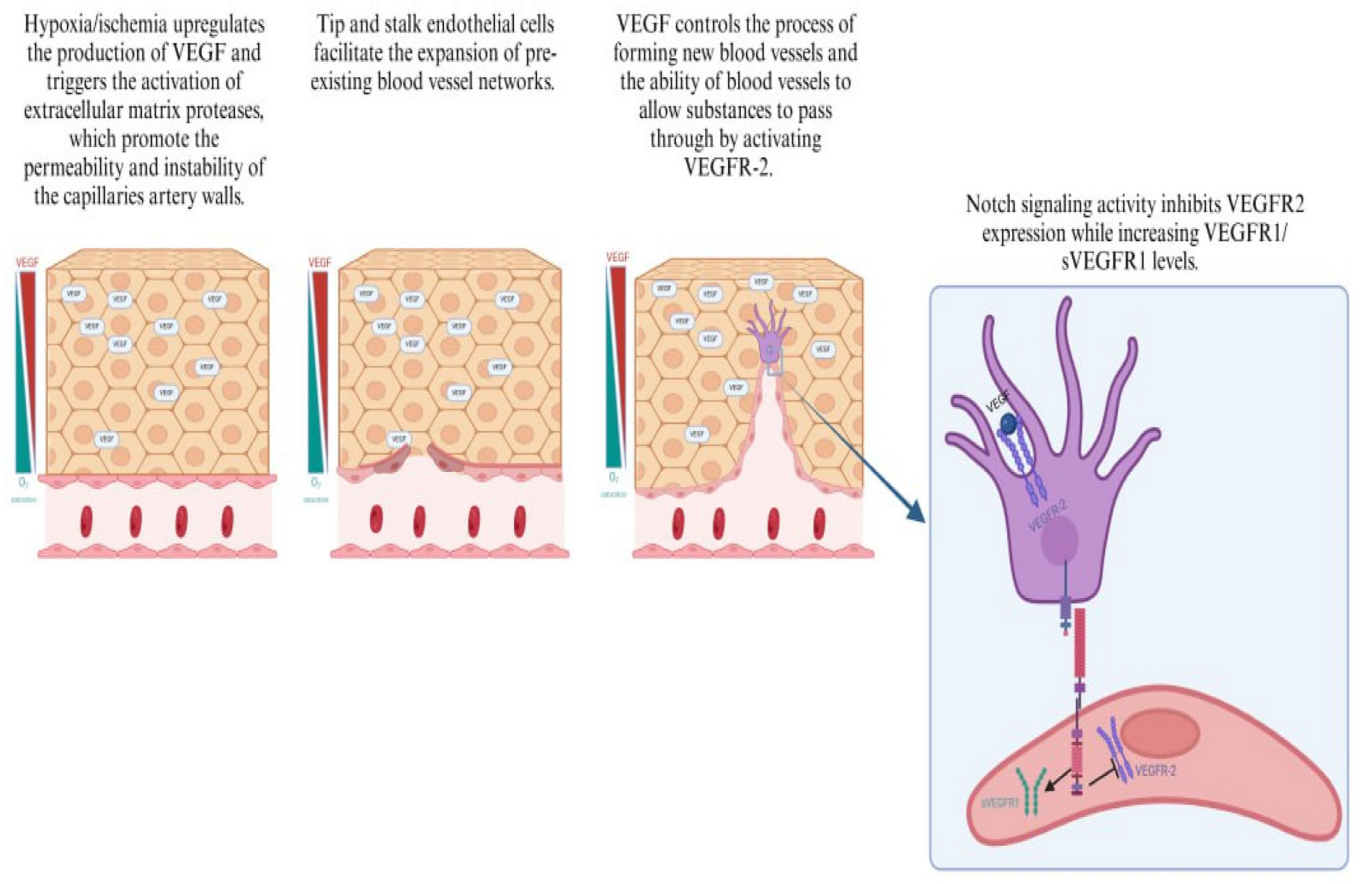
**Schematic diagram of the interaction of VEGF/VEGFR and Notch signaling cascade:** The VEGF-VEGFR system is associated with other signaling networks that regulate angiogenesis, such as the Delta (Dll4)–Notch pathway. Elevated levels of VEGF, which binds to VEGF-R2, stimulate the upregulation of Delta in a specialized kind of endothelial cell called a 'tip cell'. This, in turn, triggers a signaling pathway including Notch and leads to reduced levels of VEGF-R2 in neighboring 'stalk cells'. Activated Notch receptors on endothelial cells may influence the production of VEGF receptors in those cells, either favorably (VEGF-R1, VEGF-R3) or negatively (VEGF-R2). Notch has the ability to deliver negative feedback in order to decrease the activity of the VEGF/VEGF-R2 axis.

VEGF-A regulates blood vessel permeability via the VEGF-A/VEGFR-2 pathway. VEGF-165 also mediates vascular permeability through the natural production of platelet-activating factor (PAF) and NO [51]. VEGF enhances angiogenesis during tissue growth, regeneration as well as in response to injury or disease [52]. The VEGF family regulates a range of functions, including the development of new blood vessels, lymphocyte production, inflammation response regulation, oxidative stress damage protection, and lipid metabolism regulation [53]. These functions have significant potential for treating ischemia. VEGF-A, VEGF-B, VEGF-C, VEGF-D, VEGF-E (derived from viruses), VEGF-F (derived from snake venom), PIGF, and EG-VEGF comprise the VEGF family. Additionally, VEGF receptors consist of three types, namely VEGFR-1, VEGFR-2, and VEGFR-3. Vascular endothelial cells (VECs) predominantly express VEGFR-1 and VEGFR-2, while lymphatic endothelial cells (LECs) primarily express VEGFR-3 (Fig. 3) [54]. VEGF family controls the growth and widening of lymphatic vessels, which release inflammatory substances that have built up in the area [55].

#### 4.1.4 Fibroblast growth factors (FGFs)

FGFs are a group of proteins that attach to heparin and have important functions in the processes of angiogenesis, healing of wounds, and embryonic development. FGFs play a crucial role in the multiplication and specialization of many types of cells and tissues [[56], [57], [58]]. FGF-1 and FGF-2 have an important role in promoting the growth and arrangement of endothelial cells into tube-like formations [59]. This process, known as angiogenesis, is essential for the proper formation of new blood vessels in the body, which is necessary for tissue growth, repair, and regeneration. Thereby The ability of FGFs to promote the growth of blood vessels suggests that they could serve as effective therapeutic agents for treating ischemic conditions [60].

A new study showed that giving FGF-2 to mice with blocked leg arteries can cause new blood vessels to grow and improve blood flow. This could lead to the discovery of a treatment for CLI in humans [61]. Based on two randomized studies, it has been shown that administering FGF-1 to patients with CLI may decrease the need for amputation. Studies show FGF-1 does not improve ulcer healing or major amputation time but may reduce amputation need in CLI patients. Thus, we need more research to identify the long-term advantages and hazards of FGF-1 therapy for CLI patients and its validity as a treatment [62].

### 4.2 PRP as a source of inflammatory cytokines secretion in CLI repair

Cytokines, released by platelets, regulate the immune system [63]. Cytokines include RANTES/CCL5, PF-4/CXCL4, ENA-78/CXCL5, and NAP-2/CXCL7. Platelets use RANKL and P-selectin to interact with other cells [59,60]. Hybridoma growth factor (HGF) is a cytokine present in PRP that exhibits potent anti-inflammatory properties. HGF can change the actions of important inflammatory mediators, such as mPGES-1, cyclooxygenase (COX) families 1 and 2, and L-1β [64]. In vitro studies indicate that PRP can suppress the expression of IL-1β-induced COX-2, MMP-13, and NOS2 in chondrocytes, suggesting therapeutic potential in inflammatory conditions [61,62].

PRP contains two types of proteins called IL-1β and IL-10 [65]. IL-1β can activate certain pathways that cause inflammation, while IL-10 can block those pathways and reduce inflammation [66,67]. This means that PRP has the potential to promote healing and reduce inflammation in a variety of situations where inflammation is a contributing factor [63]. PRP therapy can inhibit the activation of the NF-κB pathway, thereby reducing the production of pro-inflammatory cytokines like IL-1β and TNF-α, thereby promoting an anti-inflammatory response [68]. PRP therapy increases catalase (CAT), glutathione S-transferase (GST), and glutathione (GSH), protecting cells from oxidative stress and damage. Moreover, PRP therapy increases BCL2 expression and decreases caspase-3 expression. These findings imply PRP therapy may boost cell survival and tissue healing [69].

### 4.3 PRP on promotion neovascularization in CLI repair

Therapeutic neovascularization is a medical strategy to boost blood flow to the lower limbs and encourage the growth of new blood vessels [70]. PRP has shown potential in supporting both angiogenesis and arteriogenesis, two critical processes in restoring blood flow to ischemic tissues. Angiogenesis involves the formation of new capillaries and small vessels, while arteriogenesis refers to the enlargement and remodeling of pre-existing collateral arteries to improve circulation in areas with limited blood supply [71]. Kim et al. found that using angiogenic-factor-enriched PRP led to faster and more extensive tissue regeneration through rapid angiogenesis during the initial healing period [72]. Natsuko Kakudo et al. demonstrated that PRP causes vascular endothelial cells to undergo proliferation, migration, and tube formation in an in vitro setting [73].

In addition to these essential growth factors, Other growth factors such as bFGF, IGF-1, and SDF-1 have also been shown to play a role in promoting angiogenesis either directly or indirectly for instance, studies have shown that bFGF stimulates the proliferation of endothelial cells, while IGF-1 enhances their survival. PRP, which has a lot of these growth factors, may help make new blood vessels grow in people who have limb ischemia, according to research [74]. VEGF and SDF-1 affect endothelial progenitor cell recruitment and homing, according to prior studies. Asahara and colleagues found that VEGF mobilizes bone marrow-derived endothelial progenitor cells to cause postnatal neovascularization [75].

De Falco et al. suggested that making SDF-1 for a short time might help stem cells move into ischemic tissue, which could lead to more neovascularization, or the growth of new blood vessels [46,47]. Shyamal et al. suggest that PRP contains a larger amount of stromal cell-derived factor-1 (SDF-1) and a smaller amount of VEGF. Through in vivo investigations, researchers have shown that the continuous release of PRP speeds up the migration of hematopoietic progenitor cells to the place damaged by ischemia. This suggests that the prolonged release of PRP stimulates the development of new blood vessels in hind limb ischemia [74].

## 5 Clinical application of PRP in limb ischemia

PRP's application in managing chronic limb ischemia, especially diabetic foot ulcers and critical limb-threatening ischemia, is evident, despite facing challenges. Recent studies have assessed the therapeutic efficacy of PRP in patients with CLI, notably those with diabetes. A study found that PRP treatment reduced ulcer size by over 90 % and limb salvage rate by 89 %, with a marginally lower rate in those with CLI [76,77]. Another comprehensive review in the American Diabetes Association compendium emphasized the expanding function of PRP in treating hard-to-heal diabetic foot ulcers. Growth factors such as VEGF and FGF promote angiogenesis and tissue regeneration, which are responsible for PRP's potential advantages. This study showed that PRP's effectiveness in wound healing and antibacterial effects is influenced by factors such as ischemia severity, comorbidities like diabetes, and methods of PRP delivery [78].

The potential of PRP in combination with other therapies such as negative pressure wound therapy (NPWT) or autologous adipose tissue grafting has been explored. A study demonstrated that PRP, when combined with adipose tissue grafting, can effectively heal lower limb ulcers within an average of 9.7 weeks. Studies reveal several challenges and limitations of PRP therapy in treating CLI, despite potential outcomes:

**Heterogeneous Responses:** Patient outcomes differ due to variations in PRP preparation techniques and the presence of comorbid conditions like diabetes or severe vascular disease.

**Lack of standardization:** Clinical trials and outcomes vary due on PRP concentration, volume, and application methods.

**Ineffectiveness in advanced ischemia**: PRP alone may not treat severe limb ischemia, especially when tissue necrosis is widespread or major arterial occlusions persist. CLI generally lacks vascular networks to trigger angiogenesis with PRP [79,80].

**Transient effects:** Angiogenesis is often temporary with PRP. Without correct integration into the existing vascular network, new blood vessels may not be stable, and PRP may lose its effects over time without additional interventions [77,81]. PRP's short-term benefits in CLI and limb salvage are becoming increasingly evident, but its long-term effects are still under investigation. In severe cases of CLI with near-total vascular occlusion, PRP may not restore blood flow without surgical revascularization, reducing PRP's long-term effectiveness [82]. Some studies have shown long-term wound closure and survival, but outcomes vary. One study found that PRP-treated chronic ulcers healed faster than standard wound care after 6 months [82]. PRP is useful; however, wounds sometimes recur or heal slowly in people with significant ischemia, showing that it is not a permanent treatment for all patients.

PRP is a promising treatment for chronic ischemia patients who have limited options. It should be part of a multimodal approach called combination therapy, including revascularization procedures when possible [83]. PRP alone causes angiogenesis and partial wound healing, but when paired with stem cells, gene therapy, or revascularization, it enhances and prolongs outcomes [82]. For instance, a clinical trial found that combining PRP and stem cells in CLI patients improved limb salvage and ulcer healing outcomes compared to either therapy alone. Combining PRP with MSCs, which possess regenerative abilities and can differentiate into various cell types, can enhance neovascularization [84].

A study found that the combination of adipose-derived stem cells (ASCs) and PRP significantly promotes angiogenesis and improves limb salvage and healing in patients with severe ischemia.

[76]. In clinical trials, this combination improved pain alleviation, ulcer healing, and limb function more than PRP or stem cells alone [79]. Animal studies demonstrated that PRP increased VEGF expression, enhancing limb perfusion and tissue survival [85]. Synoptically, more large-scale, randomized controlled trials are needed to further understand PRP's function in CLI, its long-term advantages, and combination therapy, especially in complex and severe limb ischemia.

## 6 Conclusion

Autologous blood preparation for PRP reduces disease transmission, immunogenic reactions, and cancer risk, but may cause injection site morbidity, infection, or nerve or blood vessel damage. According to the review, platelet levels above basal value facilitate tissue regeneration with minimal adverse effects. PRP composition varies across people and can also be affected by the type of preparation tool used, how long it is stored, how it is stored, and whether or not it interacts with other materials or biologics. Growth factors are crucial for angiogenesis, and organ regeneration after CLI, and their use in PRP-derived treatments has opened new avenues for treating CLI. Despite numerous positive results from animal studies, there is a lack of well-controlled human research.

## Ethical statement

This study did not involve humans or animals, so no ethical approval was required.

## Author contributions statement

The concept, design, and final approval of the manuscript for publication was done by H.M.

A.J.H, F.G., S.D., J.J.S, M.M, and M.S wrote the main manuscript text.

H.T., S.D., and S.N. prepared the work for submission by critically revising for important intellectual content.

A.A. designed the figures and Tables.

All authors read and approved the final manuscript, and are agreed on being accountable for all aspects of the work in ensuring that questions related to the accuracy or integrity of any part of the work are appropriately investigated and resolved.

## Data availability

Data sharing is not applicable.

## Declaration of competing interest

No potential conflict of interest was reported by the authors.
